# Commentary: Philippine Mental Health Act: just an act? A call to look into the bi-directionality of mental health and economy

**DOI:** 10.3389/fpsyg.2025.1513775

**Published:** 2025-05-16

**Authors:** Myles Joshua Toledo Tan, Nicholle Mae Amor Tan Maravilla

**Affiliations:** ^1^Department of Electrical and Computer Engineering, Herbert Wertheim College of Engineering, University of Florida, Gainesville, FL, United States; ^2^Department of Epidemiology, College of Public Health and Health Professions and College of Medicine, University of Florida, Gainesville, FL, United States; ^3^Biology Program, College of Arts and Sciences, University of St. La Salle, Bacolod, Philippines; ^4^Department of Natural Sciences, College of Arts and Sciences, University of St. La Salle, Bacolod, Philippines; ^5^Department of Chemical Engineering, College of Engineering and Technology, University of St. La Salle, Bacolod, Philippines; ^6^Department of Electronics Engineering, College of Engineering and Technology, University of St. La Salle, Bacolod, Philippines; ^7^Yo-Vivo Corporation, Bacolod, Philippines

**Keywords:** causal inference, econometrics, healthcare legislation, mental health, mental health economics, mental health legislation, mental health services utilization, Philippines

## Introduction

In 2021, our paper “Philippine Mental Health Act: just an act? A call to look into the bi-directionality of mental health and economy” (Maravilla and Tan, 2021) initiated a critical dialogue regarding the intricate relationship between mental health and economic factors in the context of the Philippines. We emphasized the bi-directional nature of this relationship, positing that a robust economy not only enhances mental health outcomes but that improved mental health can also drive economic growth. This foundational work was a significant advancement in the discourse surrounding mental health in the Philippines. However, with 2025 well underway, it is imperative to transition from merely discussing this relationship to actively investigating and implementing strategies that leverage this connection for policy development and economic planning.

Despite the foundational insights provided by our 2021 paper (Maravilla and Tan, 2021), there remains a notable absence of subsequent research specifically examining the mental health-economy nexus within the Philippine context. Even those studies that have referenced the original work have not studied the economic implications of mental health in the Philippines. This gap in the literature highlights the urgent need for empirical studies that explore this critical intersection, which could inform effective policy interventions and resource allocation. This lack of follow-up research may stem from various factors, potentially including limited funding dedicated to interdisciplinary studies, challenges in accessing integrated mental health and economic data, or shifting institutional priorities, which are recognized barriers in the Philippine health sector (Martinez et al., 2020; Dela Peña et al., 2024; Aldalaeen et al., 2025).

Furthermore, integrating these perspectives allows for a more holistic understanding. For example, human capital theory frames mental wellbeing as crucial for productivity (Becker, 1962; Rosen, 1976), while behavioral economics can explain how mental health challenges might impede rational economic decision-making related to work and investment in that capital (Harlé et al., 2010). Causal inference tools then provide methods to rigorously test the real-world impacts of policies derived from this integrated framework (Pearl, 2010).

## Advancing beyond initial insights: the need for deeper economic exploration

Our paper (Maravilla and Tan, 2021) outlined how mental health impacts economic factors such as labor productivity, unemployment, and overall economic stability, while also noting that economic downturns can exacerbate mental health issues. However, a more nuanced understanding of the mechanisms underlying this relationship is necessary. To facilitate real change, it is essential to refine analyses of the interactions between mental health and economic variables, employing interdisciplinary approaches that incorporate economic theory, public health insights, and behavioral science.

## Causal inference: from correlation to causality

To advance our understanding from mere correlation to establishing causality, it is crucial to utilize causal inference methodologies (Pearl, 2010) such as difference-in-differences (DiD) (Angrist and Krueger, 1999) and instrumental variable (IV) (Wright, 1928; Heckman, 2008) techniques. These methodologies can help disentangle the complex relationships between mental health and economic outcomes, providing vital insights for targeted interventions. For instance, applying DiD to assess the impact of mental health interventions post-implementation of the Philippine Mental Health Act could reveal the true effects of mental health improvements on productivity and employment rates, thereby informing future policy decisions (Wang et al., 2024). Similarly, an IV approach could, for instance, use differential regional access to newly introduced telehealth services as an instrument to estimate the causal effect of improved mental healthcare access on local employment rates, controlling for other confounding factors. Combining methods like fixed effects with IV has proven useful in assessing psychosocial factors and mental health in other contexts (Milner et al., 2018).

## Behavioral economics: bridging the gap between policy and human decision-making

Behavioral economics presents another promising avenue for enhancing mental health policy. Our paper (Maravilla and Tan, 2021) highlighted how mental health issues can impair decision-making processes, but a deeper exploration into how these issues distort economic choices is warranted. For example, conditions like depression may lead to poor financial decisions (Harlé et al., 2010), while anxiety can deter individuals from seeking necessary healthcare (Horenstein and Heimberg, 2020). By integrating behavioral economics into mental health programs, innovative strategies can be developed to encourage healthier economic behaviors, such as incentivizing mental health check-ups or improving access to mental health services through technology.

## Human capital theory: mental health as an economic investment

Viewing mental health through the lens of human capital theory (Becker, 1962; Rosen, 1976) can further bolster the economic argument for investing in mental health services. This perspective positions mental health as a vital component of human capital, which directly influences labor market productivity and innovation. By quantifying the economic contributions of mental health to human capital, policymakers can justify increased investments in mental health infrastructure as a strategic economic initiative, promoting sustained economic development through a healthier workforce (World Health Organization, 2020).

## Cost-benefit analysis: quantifying the economic return on mental health investments

The need for policy reform is evident, yet it is essential to conduct comprehensive cost-benefit analyses (CBA) to quantify the economic returns on mental health investments. Such analyses can elucidate the long-term savings associated with improved mental health, including reduced healthcare costs and increased labor force participation. In the context of the Philippines, where healthcare resources are limited, demonstrating the economic advantages of mental health investments could persuade policymakers to prioritize mental health alongside other pressing economic issues.

## Machine learning and causal pathways: uncovering hidden patterns

Recent advancements in data science, particularly machine learning (ML), offer new opportunities to explore the mental health-economy nexus. ML can identify hidden patterns within extensive datasets, enabling researchers to uncover causal pathways that traditional methods may overlook (Kaddour et al., 2022). In the Philippines, causal ML could be employed to analyze diverse data sources, such as employment records and healthcare utilization, to assess how mental health interventions impact economic variables like workforce participation.

Practically, this could involve applying algorithms like causal forests or deep learning models to large-scale longitudinal datasets integrating anonymized health records, Philippine Health Insurance Corporation (PhilHealth) claims, employment data [e.g., Social Security System (SSS) contributions], and possibly survey data on wellbeing. Uncovering complex non-linear relationships or identifying specific subgroups most impacted by interventions would be key goals. This approach could facilitate more targeted and effective policy interventions that maximize both mental health and economic outcomes.

A significant prerequisite for leveraging these advanced methodologies is addressing the state of data infrastructure in the Philippines. Challenges related to data fragmentation across different government agencies (e.g., Department of Health [DOH], Department of Labor and Employment [DOLE], Philippine Statistics Authority), varying data quality, and ensuring robust data privacy and sharing protocols need to be systematically tackled to enable the large-scale integrated analysis that is required.

## Economic shocks and mental health: building resilience through policy

Our paper (Maravilla and Tan, 2021) identified economic crises as a significant factor exacerbating mental health issues in the Philippines. In light of recent global crises, such as the COVID-19 pandemic, understanding the mental health impacts of economic shocks has become increasingly urgent (Silva et al., 2018; Talamonti et al., 2023). Employing modern econometric techniques can help estimate the mental health toll of economic disruptions, guiding the development of responsive economic policies that address public mental health needs. Proactive policy measures, including social safety nets, can mitigate the mental health impacts of economic downturns, thereby enhancing overall societal resilience (Wahlbeck and McDaid, 2012).

## Discussion: toward an integrated mental health-economic policy

As we reflect on the discussions initiated by the Philippine Mental Health Act, it is clear that the time has come to build upon these insights by applying rigorous, data-driven economic tools. Despite the potential relevance of this topic, no research has yet specifically examined the bi-directional relationship between mental health and the economy in the Philippine context, and even those articles citing our 2021 paper (Maravilla and Tan, 2021) have not explored this further. By leveraging causal inference techniques, behavioral economics, human capital theory, cost-benefit analysis, and machine learning, policymakers in the Philippines can design more effective interventions that not only enhance mental health outcomes but also promote sustainable economic growth.

Moreover, understanding the bi-directional relationship requires examining the specific socio-economic and cultural landscape of the Philippines. Factors such as significant regional disparities in access to both economic opportunities and mental health services, prevalent cultural beliefs and stigma surrounding mental health, and the unequal distribution of resources across the archipelago likely moderate this relationship in unique ways that demand context-specific policy approaches (Martinez et al., 2020; McDaid et al., 2008; World Health Organization, 2021).

Learning from international experiences and case studies where other nations, including low- and middle-income countries like South Africa and Uganda, have integrated mental health considerations into primary care and economic policy (Petersen et al., 2011) could also provide valuable comparative insights and adaptable models for the Philippines.

Translating these insights into policy also necessitates acknowledging the specific challenges faced by policymakers in the Philippines. These may include navigating budget constraints within the national health and economic agenda, where mental health historically receives limited funding (Rogayan and De Guzman, 2024), ensuring effective inter-agency coordination between executive departments like the DOH, Department of Social Welfare and Development, DOLE, and the National Economic and Development Authority; and aligning mental health initiatives with existing economic development plans and social protection programs such as the Pantawid Pamilyang Pilipino Program (4Ps) (World Bank, 2024). Demonstrating cost-effectiveness becomes crucial in this political economy.

Furthermore, effective and culturally appropriate policy design and implementation must actively involve civil society organizations, non-governmental organizations, and local communities, whose voices are crucial for ensuring interventions are accepted, accessible, and meet genuine needs on the ground (World Health Organization, 2021).

Crucially, investing in mental health should be framed not only as an economic strategy but also as a fundamental aspect of social inclusion, aligning with the rights-based approach of the Mental Health Act. Policies must prioritize reaching vulnerable and marginalized populations, including persons with disabilities, those in geographically isolated and disadvantaged areas, and informal sector workers, ensuring that the economic and wellbeing benefits are equitably shared and reducing the social exclusion often faced by those with mental health conditions. The challenge now lies in translating theoretical discussions into actionable insights, ensuring that mental health is recognized as a crucial component of economic development.
